# Packing Density Governs Tobacco Quality Through Microbial Community Assembly and Metabolic Reprogramming

**DOI:** 10.3390/microorganisms14071454

**Published:** 2026-07-01

**Authors:** Bo Fu, Hui Zhong, Tao Liu, Xinying Li, Pengwei Yao, Yunpeng Fu, Jing Wang

**Affiliations:** The Key Lab of National Tobacco Cultivation, College of Tobacco Sciences, Henan Agricultural University, Zhengzhou 450002, China

**Keywords:** *Nicotiana tabacum* L., flue-cured tobacco, packing density, microbial community, aroma compounds, community assembly

## Abstract

Packing density regulates the microenvironment of tobacco (*Nicotiana tabacum* L.) fermentation and may thereby influence microbial activity and product quality. However, its effects on microbial community assembly and quality formation remain poorly understood. This study aimed to clarify how packing density affects flue-cured tobacco quality by shaping microbial communities, functional potential, and ecological interactions. Here, we investigated the effects of three packing densities (60%, 70%, and 80%) on chemical components, aroma compounds, microbial community structure, functional potential, co-occurrence networks, and assembly mechanisms of flue-cured tobacco (cv. Piaohe No. 2) after 10 days of fermentation. Moderate density (70%) achieved the most balanced chemical profile, with appropriate nicotine retention, potassium/chlorine ratio, and sugar/nicotine balance. T70 also exhibited the highest levels of total esters, total ketones, and β-ionone, key contributors to fruity, floral, and woody aromas. Microbial analysis revealed that T70 supported the highest diversity and was characterized by the enrichment of aroma-related bacterial taxa, including *Bacillus* and lactic acid bacteria, as well as the fungal genus *Pichia*. In contrast, T60 favored aerobic nicotine degraders, whereas T80 selected for obligate anaerobes associated with off-odor production. Functional predictions and network analysis showed that T70 upregulated fatty acid and carotenoid biosynthesis pathways and exhibited the highest modularity, indicating a compartmentalized, functionally complementary community. Neutral model fitting revealed increasing stochasticity with density, with T70 displaying a mixed assembly regime. Collectively, our findings show that packing density influences tobacco quality by regulating microbial community composition, functional potential, network interactions, and assembly processes. These results provide a scientific basis for optimizing packing density in tobacco processing.

## 1. Introduction

Sun-cured tobacco (*Nicotiana tabacum* L.) has long been an important processing method, but it suffers from several limitations. The process is highly dependent on weather conditions, leading to inconsistent product quality [1]. It also offers limited control over temperature and humidity, often resulting in incomplete fermentation and undesirable harshness [2]. Prolonged outdoor exposure increases the risk of contamination by undesirable microorganisms, compromising both safety and sensory quality [3]. These drawbacks have driven the search for more reliable alternatives. Flue-curing has emerged as a superior alternative, offering several distinct advantages. Unlike sun-curing, flue-curing is conducted in enclosed chambers where temperature and humidity can be precisely controlled, enabling consistent fermentation outcomes regardless of external weather [4]. This controlled environment maximizes the degradation of undesirable compounds while preserving desirable flavor precursors [5]. Moreover, flue-curing significantly reduces processing time and the risk of microbial contamination. These advantages have made flue-curing the predominant method for high-quality tobacco in many regions.

Fermentation is a critical step that determines the ultimate quality of flue-cured tobacco. Unlike simple drying or curing, fermentation is an active biotransformation process in which indigenous microbial communities and endogenous enzymes work synergistically to reshape the chemical and sensory properties of tobacco leaves [6,7]. Previous studies have shown that fermentation promotes the degradation of undesirable constituents, particularly nicotine and other nitrogenous compounds associated with physiological harshness and irritation [8]. It also facilitates the formation of desirable metabolites, including reducing sugars, organic acids, and volatile aroma compounds [9]. These biochemical transformations directly affect smoke smoothness, aroma complexity, combustibility, and overall product acceptability, highlighting the central importance of fermentation in tobacco quality improvement.

The microbial community is the primary engine driving these transformations. Different microbial guilds play distinct yet complementary functional roles. Aerobic bacteria are efficient nicotine degraders and producers of extracellular hydrolases that liberate substrates from complex macromolecules [8]. Yeasts generate higher alcohols and esters from amino acids via the Ehrlich pathway, contributing floral and fruity notes [10]. Lactic acid bacteria not only lower pH through organic acid production but also directly catalyze ester formation, adding sweetness and complexity to the aroma profile [10]. Importantly, these guilds do not operate in isolation. They engage in metabolic cross feeding and functional complementarity, meaning that the structure of the microbial community directly governs the flux through these pathways and, ultimately, the quality of the fermented product [11]. However, in current tobacco fermentation practice, the regulation of packing density still relies largely on empirical experience, and its microbiological and ecological basis remains insufficiently understood. Therefore, understanding how processing parameters regulate microbial community assembly and function is essential for improving the predictability and optimization of tobacco fermentation.

Despite the recognized importance of fermentation, the effect of a fundamental processing parameter, packing density, remains surprisingly understudied. Packing density governs multiple microenvironmental factors simultaneously, including oxygen availability, moisture retention, heat dissipation, and physical space for microbial dispersal and interaction [12]. Previous studies have largely focused on other fermentation parameters such as temperature, humidity, and duration, while the influence of packing density on microbial community assembly, functional metabolism, and tobacco quality has received little attention [13,14]. Moreover, existing research has primarily described microbial compositional changes without probing the ecological processes or network interactions that underlie these shifts, leaving a critical gap in our mechanistic understanding [15].

To address this gap, we selected the tobacco cultivar Piaohe No. 2 as our experimental material and established three packing density treatments (60%, 70%, and 80%). Because tobacco cultivar can influence leaf chemical composition, microbial colonization, and fermentation performance, the findings of this study should be interpreted within the context of this specific cultivar. The aims of this study were to (1) characterize the effects of packing density on key chemical components and aroma compounds; (2) elucidate the taxonomic and functional shifts in microbial communities underlying these quality differences; (3) reveal how packing density governs microbial community assembly processes and ecological interactions. Our findings establish a mechanistic link between packing density and tobacco quality through microbial ecology, providing a scientific basis for optimizing packing density in tobacco processing.

## 2. Materials and Methods

### 2.1. Plant Materials and Fermentation Conditions

The fermentation experiment was conducted at the Key Lab of National Tobacco Cultivation, Henan Province, China (34.77° N, 113.67° E), from 18 May to 27 May 2025. During the experimental period, the daily mean ambient temperature ranged from 20 to 29 °C, and the ambient relative humidity ranged from 45% to 65%. Tobacco leaves of the cultivar Piaohe No. 2 were used as the experimental material. The leaves were preconditioned to an initial moisture content of 18–22%. Cylindrical oak barrels with a bottom diameter of 21 cm and a height of 27 cm (approximately 9347 cm^3^) were used as fermentation vessels. For each treatment, 2500 g of leaves were uniformly spread into the barrel. The barrel lid was then placed directly on the tobacco leaves, and sandbags weighing 250 g, 350 g, or 450 g were placed on the lid to apply different levels of compression, thereby achieving the 60%, 70%, and 80% packing-density treatments, respectively.

In this study, the packing-density treatments refer to relative packing compactness. Relative packing compactness was defined according to the compression level used in the processing design, using the maximum compacted state achievable for the same mass of tobacco leaves in the same barrel as the reference. Specifically, the 250 g sandbag represented the low-compaction treatment (T60), the 350 g sandbag represented the moderate-compaction treatment (T70), and the 450 g sandbag represented the high-compaction treatment (T80). Fermentation was conducted under controlled conditions at 40 °C and 80% relative humidity for 10 days. Upon completion of fermentation, samples were collected and stored for subsequent chemical, aroma, and microbiological analyses.

### 2.2. Determination of Major Chemical Constituents

The major chemical constituents of tobacco leaves were determined following standard protocols. All determinations were performed in six replicates, and results were expressed on a dry weight basis. Total nitrogen was measured by the Kjeldahl method using a Kjeltec 8400 fully automatic Kjeldahl analyzer (Foss, Hillerød, Denmark). Nicotine was determined by ultraviolet spectrophotometry using a UV-2600 spectrophotometer (Shimadzu, Kyoto, Japan).

Total sugar and reducing sugar were quantified using the continuous flow analysis method on an AA3 continuous flow analyzer (SEAL Analytical, Norderstedt, Germany).

Potassium was determined by flame photometry using an M410 flame photometer (Sherwood, Cambridge, UK). Chlorine was measured by automatic potentiometric titration using a T960 automatic titrator (Hanon, Shanghai, China). Because potassium and chlorine were quantified using different analytical methods, potential method-specific differences in accuracy may influence the calculated potassium/chlorine ratio. To minimize method-related uncertainty, both instruments were calibrated with standard solutions before analysis, and blank controls and replicate measurements were included for quality control. The reducing sugar/total sugar ratio, potassium/chlorine ratio, and sugar/nicotine ratio were subsequently calculated from the measured values.

### 2.3. Determination of Aroma Compounds

Aroma compounds were analyzed using gas chromatography-mass spectrometry (GC-MS). Samples were extracted with dichloromethane by simultaneous distillation extraction. Separation was performed on an HP-5MS capillary column (30 m × 0.25 mm × 0.25 μm) with helium as carrier gas at 1.0 mL/min. The oven temperature was programmed from 50 °C to 250 °C. The mass spectrometer was operated in electron impact mode (70 eV), and compounds were identified using the NIST library. Quantification was performed using n-hexadecane as the internal standard.

### 2.4. Microbial Sequencing and Analysis

Total genomic DNA was extracted from tobacco leaf samples using a DNA extraction kit (Omega Bio-Tek, Norcross, GA, USA) according to the manufacturer’s instructions. DNA concentration and purity were assessed using a NanoDrop spectrophotometer (Thermo Fisher, Waltham, MA, USA).

For bacterial community analysis, the V3–V4 region of the bacterial 16S rRNA gene was amplified. For fungal community analysis, the ITS1 region was amplified. PCR products were purified, quantified, and sequenced on an Illumina NovaSeq 6000 platform (Illumina, San Diego, CA, USA) using paired-end 250 bp reads. Raw sequencing data were processed using QIIME2 (v. 2022.2) [16]. Reads were demultiplexed, quality-filtered, and denoised using the DADA2 pipeline to generate amplicon sequence variants (ASVs). Bacterial taxonomy was assigned against the SILVA database (v. 138), and fungal taxonomy against the UNITE database (v. 8.0). Functional potential was predicted using PICRUSt2 (v. 2.5.0). KEGG pathway enrichment analysis was performed to identify differentially enriched pathways between treatments.

### 2.5. Co-Occurrence Network and Neutral Community Model Analysis

Co-occurrence networks were constructed to explore microbial interactions under different packing densities. Spearman correlation coefficients were calculated based on the relative abundance of microbial genera using the *Hmisc* package in R software (v. 4.2.0). Only correlations with |*r*| > 0.8 and *p* < 0.05 were considered statistically significant [17]. Networks were visualized using Gephi (v. 0.9.2), and topological parameters including nodes, edges, average degree, graph density, clustering coefficient, and modularity were calculated. Keystone taxa were identified using Zi-Pi analysis, classifying nodes as peripherals, connectors, module hubs, or network hubs [18].

The neutral community model (NCM) was fitted to quantify the relative contribution of stochastic processes to community assembly [19]. The model was implemented using the *ncm* function in R, and the fitting quality was assessed by R^2^. The migration rate (m) and metacommunity size (Nm) were estimated. The normalized stochasticity ratio (NST) was further calculated using the *NST* package in R to evaluate the balance between stochastic and deterministic assembly processes, with NST > 0.5 indicating stochastic dominance.

### 2.6. Statistical Analysis

All statistical analyses were performed using R software (v. 4.2.0). Differences in chemical components, aroma compounds, and alpha diversity indices among treatments were assessed using one-way analysis of variance (ANOVA) followed by Tukey’s honestly significant difference (HSD) post hoc test using the *stats* package and the *multcomp* package in R. Beta diversity was evaluated using principal coordinate analysis (PCoA) based on Bray–Curtis distances using the *vegan* package. Analysis of similarities (ANOSIM) was performed using the *vegan* package to test for significant differences in community structure. Differential abundance of taxa was identified using LEfSe analysis with a linear discriminant analysis (LDA) threshold of 2.0 (*p* < 0.05). Data visualization was performed using the *ggplot2* package. All experiments were performed with six replicates.

## 3. Results and Discussion

### 3.1. Packing Density Modulates Chemical Transformation During Tobacco Fermentation

**Figure 1:**
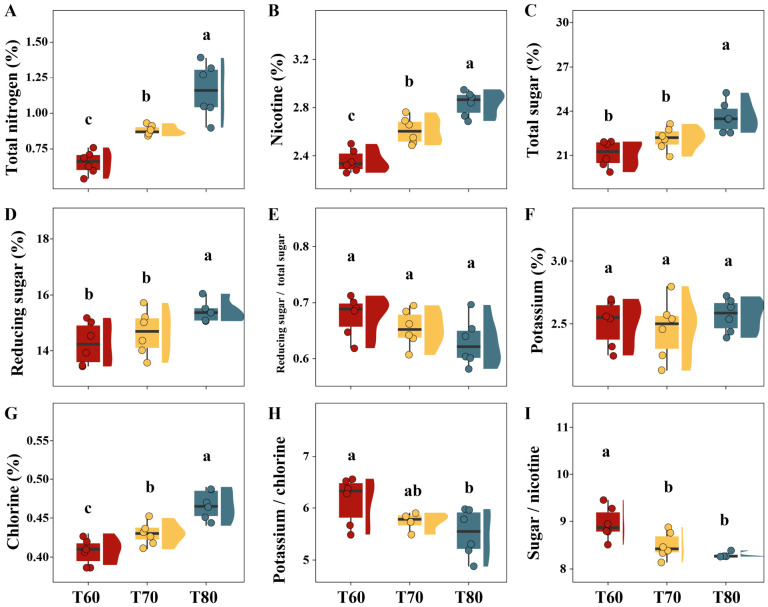
Effects of different packing densities on chemical components of flue-cured tobacco leaves. (**A**) Total nitrogen, (**B**) nicotine, (**C**) total sugar, (**D**) reducing sugar, (**E**) reducing sugar/total sugar ratio, (**F**) potassium, (**G**) chlorine, (**H**) potassium/chlorine ratio, and (**I**) sugar/nicotine ratio. Data are presented as mean ± SD (n = 6). Different lowercase letters indicate significant differences among treatments.

The chemical composition of tobacco leaves under three packing densities is presented in Figure 1, which collectively reflects the extent of biochemical transformations during fermentation. Specifically, the total nitrogen content increased progressively with packing density, with T80 exhibiting the highest value, followed by T70 and T60, and all pairwise comparisons were statistically significant (*p* < 0.05) (Figure 1A). Nicotine content followed an identical pattern (*p* < 0.05) (Figure 1B). Nicotine is one of the most important alkaloids in tobacco, because it largely determines physiological strength, sensory impact, and smoking satisfaction. These findings indicate that lower packing densities promote more extensive degradation of nitrogenous compounds, including nicotine. The underlying mechanism likely involves enhanced oxygen availability in loosely packed tobacco (T60), which stimulates aerobic microorganisms that utilize nicotine as a carbon and nitrogen source. Previous research has demonstrated that microorganisms belonging to the normal microflora of tobacco leaves exhibit particularly rapid denitrifying effects when provided with adequate oxygen availability, such as *Pseudomonas* and *Micrococcus* species [20].

Conversely, high-density packing (T80) restricts oxygen diffusion, creating hypoxic conditions that suppress these aerobic degraders, thereby retaining higher nicotine content. From a sensory perspective, nicotine is the primary source of physiological strength and smoking satisfaction [21]. Excessive reduction as seen in T60 may lead to an unsatisfying or hollow smoking experience, particularly for habitual smokers. Conversely, the high nicotine retention in T80 could impart harshness, throat irritation, and bitterness. Thus, T70 achieves an optimal balance, reducing nicotine sufficiently to mitigate harshness while preserving adequate physiological impact.

Sugars are key quality-related compounds in flue-cured tobacco, as they contribute to smoke sweetness and serve as important precursors for Maillard reaction-derived aroma compounds. In this study, total sugar and reducing sugar contents both followed the order T80 > T70 > T60, with T80 showing significantly higher values than T70 and T60 (*p* < 0.05), while the difference between T70 and T60 was not statistically significant (Figure 1C,D). This pattern suggests that carbohydrate consumption during fermentation is density-dependent, with more extensive sugar utilization occurring under lower-density conditions. Aerobic microbial respiration and enzymatic hydrolysis are the primary drivers of sugar depletion, as sugars are oxidized to CO_2_ and water or converted into organic acids, alcohols, and esters [22]. The comparable sugar levels between T70 and T60 indicate that moderate density achieves near-maximal sugar transformation efficiency, whereas T80 suffers from insufficient microbial activity due to oxygen limitation.

Importantly, the reducing sugar/total sugar ratio showed no significant differences among the three treatments (*p* > 0.05) (Figure 1E). This stability suggests that while absolute sugar content varies with packing density, the quality of the sugar profile remains robust across the tested density range, specifically the proportion of fermentable reducing sugars that contribute directly to sweetness and Maillard reaction potential. Residual sugars contribute to smoke sweetness and serve as precursors for Maillard-derived aroma compounds [23]. Excessive sugar depletion (T60) may reduce these precursors, leading to thinner smoke flavor, while insufficient consumption (T80) may result in a green profile. T70 preserves sufficient sugar-derived sweetness while achieving meaningful transformation, representing an optimal compromise.

Potassium content remained remarkably stable across all treatments (*p* > 0.05) (Figure 1F), suggesting that its retention is governed primarily by the initial leaf composition and is largely unaffected by fermentation conditions. This stability is noteworthy because potassium is a critical determinant of tobacco combustibility, influencing the rate of coal formation, ash cohesiveness, and the completeness of combustion [24]. In contrast, chlorine content showed a density-dependent increase, with T80 > T70 > T60, and the difference between T80 and T60 was statistically significant (*p* < 0.05).

Chlorine is a volatile element that can be lost during fermentation through the volatilization of hydrogen chloride or organic chlorinated compounds [25]. The density-dependent chlorine reduction likely reflects enhanced gas exchange and moisture loss under better-aerated conditions in T60 and T70, which facilitate the escape of chlorine-containing volatiles. Conversely, the high-density environment of T80 restricts gas exchange, traps moisture and volatiles within the tobacco matrix, and thereby retains higher chlorine content (Figure 1G). Consequently, the potassium/chlorine ratio, a critical quality indicator for tobacco combustibility, ash cohesion, and smoke character, followed the inverse order T60 > T70 > T80, with T60 significantly higher than T80 (*p* < 0.05) (Figure 1H).

Generally, potassium and the K/Cl ratio are positively correlated with combustibility, while chlorine content is negatively correlated [26]. A high K/Cl ratio translates to better ash cohesion and lower tar delivery per puff [27]. The K/Cl ratio of T70 (5.73) exceeds the minimum threshold of 4.0 required for acceptable combustibility, indicating clean-burning tobacco. Although T60 exhibits a superior ratio (6.15), this advantage must be weighed against its lower nicotine and sugar content, which may compromise sensory quality.

The sugar/nicotine ratio is widely recognized as a critical determinant of smoking quality, with values between 8 and 12 generally considered optimal for flue-cured tobacco [28]. All three treatments fell within this desirable range. T60 exhibited the highest ratio, while the difference between T70 and T80 was not significant (Figure 1I). The higher ratio in T60 reflects the combined effect of more extensive sugar consumption and even more pronounced nicotine degradation, resulting in a sweeter, milder character. However, excessively high sugar/nicotine ratios (>10) are sometimes associated with bland, unsatisfying smoke in certain markets. Therefore, the moderate ratio of T70 may be advantageous from a commercial perspective, providing a balanced interplay between sweetness and physiological impact. From a product development standpoint, T70′s sugar/nicotine profile would appeal to a broad range of consumers, from those seeking a balanced, all-day smoking experience to those who prefer a milder, less irritating product.

### 3.2. Density-Dependent Aroma Compound Formation During Tobacco Fermentation

**Figure 2:**
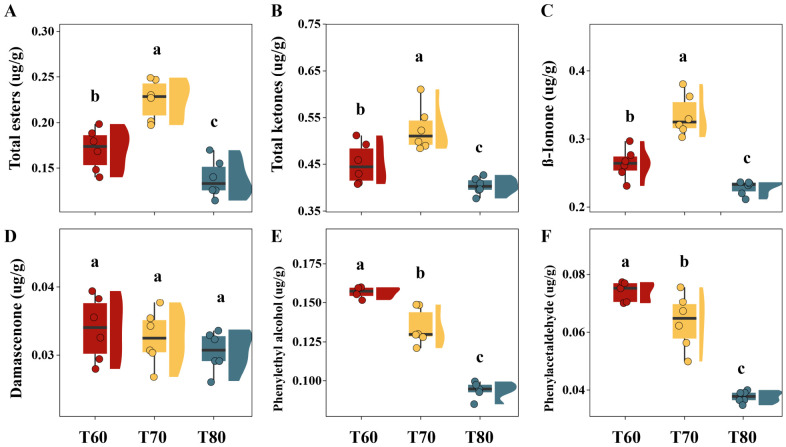
Effects of different packing densities on aroma compounds of flue-cured tobacco leaves. (**A**) Total esters, (**B**) total ketones, (**C**) β-Ionone, (**D**) damascenone, (**E**) phenylethyl alcohol, (**F**) phenylacetaldehyde. Data are presented as mean ± SD (n = 6). Different lowercase letters indicate significant differences among treatments.

The aroma compound profiles of tobacco leaves under three packing densities are presented in Figure 2. Total esters are primarily responsible for fruity and sweet aromas, which followed the order T70 > T60 > T80 with all pairwise comparisons being statistically significant (*p* < 0.05) (Figure 2A). Total ketones, which contribute to floral, woody, and tobacco-typical notes, exhibited an identical pattern (Figure 2B). These findings clearly demonstrate that moderate packing density (T70) maximizes the accumulation of both ester and ketone aroma compounds.

Esters are typically produced by esterification reactions involving alcohols and organic acids, catalyzed by microbial esterases or occurring spontaneously during fermentation [29]. Yeasts like *Pichia* and *Hanseniaspora* along with lactic acid bacteria including *Lactococcus* and *Leuconostoc* are known to produce substantial quantities of esters during fermentation [30,31]. The high ester content in T70 suggests that this density optimally supports the growth and metabolic activity of these aroma-producing microorganisms. In contrast, the low ester content in T80 reflects the suppression of aerobic and facultative microbes under oxygen-limited conditions, while the intermediate level in T60, possibly due to excessive volatilization or further metabolism of esters under highly aerobic conditions [32].

β-Ionone is a key carotenoid-derived norisoprenoid responsible for violet, woody, and berry-like aromas, which followed the same pattern as total ketones (Figure 2C). This compound is of particular importance due to its extremely low odor detection threshold (approximately 5–10 ppb) and its substantial contribution to the characteristic aroma of high-quality flue-cured tobacco [33]. The markedly higher β-ionone content observed in the T70 group strongly indicates that moderate planting density optimizes the oxidative cleavage of carotenoid precursors. This biochemical process is catalyzed by carotenoid cleavage dioxygenases derived from microorganisms [34]. Carotenoid degradation is strictly oxygen-dependent, which explains the low levels observed in T80 under oxygen-limited conditions. However, T60, despite having the highest oxygen availability, did not achieve the highest β-ionone levels. This counterintuitive finding suggests that excessive aeration may lead to over-oxidation of β-ionone to downstream compounds or may suppress the specific microbial taxa responsible for carotenoid cleavage. Indeed, certain carotenoid-degrading bacteria and fungi prefer microaerophilic conditions rather than fully aerobic environments [35].

Thus, T70′s moderate density likely creates the optimal oxygen tension for both the growth of carotenoid-degrading microorganisms and the stability of the β-ionone product.

Damascenone is another carotenoid-derived norisoprenoid that imparts honey, fruity, and sweet aromas, showing no statistically significant differences among treatments (*p* > 0.05) (Figure 2D). One possible explanation is that damascenone can be generated through multiple pathways, including direct carotenoid degradation and alternative routes from glycosidically bound precursors, which may be differentially affected by oxygen availability [36]. The consistently high damascenone levels across all treatments indicate that even suboptimal densities (T60 and T80) support sufficient formation of this important aroma compound, which is advantageous from a quality assurance perspective.

Phenylethyl alcohol is a rose- and honey-scented aromatic alcohol derived from the phenylpropanoid pathway, exhibiting a decreasing trend with increasing packing density (Figure 2E). Phenylacetaldehyde, which contributes hyacinth-like sweet and floral aromatic characteristics, exhibited a consistent accumulation pattern across treatments (Figure 2F). These results demonstrate that lower packing densities promote the accumulation of phenylpropanoid-derived aroma compounds. Both phenylethyl alcohol and phenylacetaldehyde are primarily synthesized via the Ehrlich pathway from L-phenylalanine, a process carried out by yeasts and certain bacteria [37,38]. This pathway involves transamination of phenylalanine to phenylpyruvic acid, followed by decarboxylation and reduction [39]. The higher levels of these compounds in T60 suggest that the highly aerobic conditions favor the activity of phenylalanine-utilizing microorganisms, such as *Saccharomyces cerevisiae* and *Pichia* species, which are known to produce substantial quantities of these aroma compounds under aerobic conditions [40,41].

However, from a sensory quality perspective, the optimal level of phenylethyl alcohol and phenylacetaldehyde is a matter of balance. Moderate levels contribute pleasant floral and sweet notes, but excessive concentrations can impart an overpowering, perfume-like character that may be perceived as artificial or cloying. Therefore, while T60 produced the highest absolute amounts, T70′s intermediate levels (0.13 and 0.06) may actually be closer to the sensory optimum, providing sufficient floral sweetness without risking off-notes.

**Figure 3:**
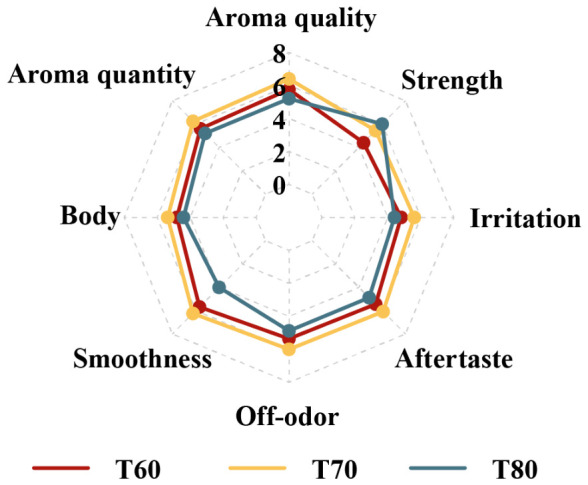
Sensory evaluation of flue-cured tobacco under different packing densities. Radar plot of eight sensory attributes (9-point scale). Higher scores indicate better quality except for strength (higher score = stronger physiological impact). For off-odor and irritation, higher scores represent fewer off-odors and less irritation, respectively.

To further verify the practical implications of these chemical and aroma differences, a sensory evaluation was performed in this study (Figure 3). The T70 treatment yields the highest scores in aroma quality, smoothness, off-odor, aftertaste, and irritation, indicating superior overall smoking quality. These sensory findings are consistent with the chemical and aroma compound analytical results, collectively confirming that moderate packing density (T70) optimizes the balance between physiological strength and smoking smoothness while maximizing aroma quality.

### 3.3. Packing Density Drives Microbial Community Assembly and the Enrichment of Aroma-Producing Taxa

**Figure 4:**
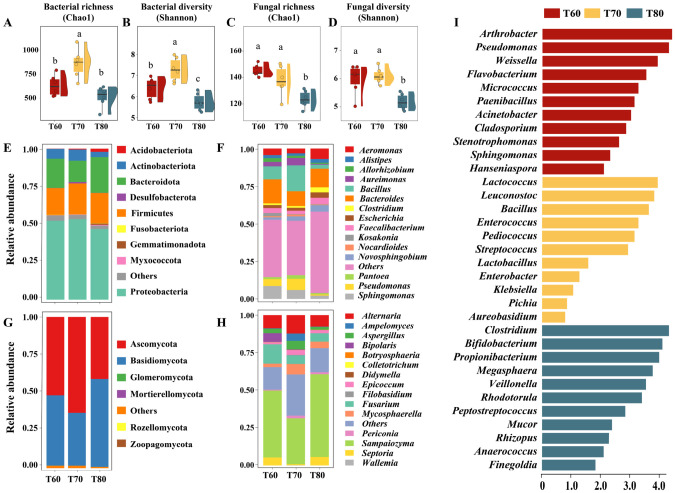
Microbial community diversity and composition under different packing densities. (**A**,**B**) Chao1 and Shannon indices of bacterial communities. (**C**,**D**) Chao1 and Shannon indices of fungal communities. Different lowercase letters indicate significant differences among treatments. (**E**,**F**) Relative abundance of bacterial and fungal communities at the phylum level. (**G**,**H**) Relative abundance of bacterial and fungal communities at the genus levels. (**I**) LEfSe analysis showing differentially abundant microbial taxa among treatments (LDA score > 2.0, *p* < 0.05).

To elucidate the microbiological mechanisms underlying density-dependent variations in chemical profiles and aromatic characteristics, this study characterized the bacterial and fungal community structures across three packing density treatments. The Shannon and Chao1 indices exhibited similar patterns for both bacterial and fungal communities (Figure 4A–D), with the highest values observed in T70. For bacterial communities, both species richness and community diversity were significantly lower in the T60 group relative to T70, while fungal community parameters showed no significant differences between the two groups. The T80 treatment consistently presented the lowest alpha-diversity values for both microbial communities. Collectively, these findings demonstrate that moderate packing density (T70) sustains the most diverse and well-structured microbial assemblages, whereas high packing density reduces microbial diversity via the competitive dominance of a small number of anaerobic and stress-tolerant microbial taxa. This microbial distribution pattern conforms to the intermediate disturbance hypothesis, which posits that moderate environmental stress maximizes diversity by preventing competitive exclusion while maintaining habitat heterogeneity [42].

At the phylum level, the bacterial community across all treatments was predominated by Proteobacteria, followed by Bacteroidota and Firmicutes (Figure 4E). Specifically, the relative abundances of Proteobacteria and Firmicutes peaked in the T70 treatment, while Bacteroidota was lowest in T70. The elevated Firmicutes abundance in T70 is particularly notable, as this phylum encompasses many genera known for aroma production (e.g., *Bacillus*, *Lactococcus*, *Leuconostoc*) and polysaccharide degradation [43,44]. The reduced Bacteroidota abundance observed in T70 may result from the diminished competitive fitness of this phylum under the unique oxygen and moisture microenvironments formed under moderate packing density. Additionally, the relative abundance of Acidobacteriota increased gradually with rising packing density, following the order of T60 < T70 < T80. Members of the Acidobacteriota are generally oligotrophic microorganisms adapted to low-oxygen and acidic microhabitats [45]. Their enrichment in T80 likely reflects the accumulation of acidic fermentation byproducts and reduced oxygen availability in high-density packing. For fungi, Ascomycota and Basidiomycota dominated all treatments, together accounting for more than 95% of the total sequences (Figure 4F). Ascomycota was highest in T70, while Basidiomycota was lowest in T70.

At the genus level, bacterial *Aeromonas*, *Bacteroides*, *Bacillus*, and *Sphingomonas* were dominant across treatments (Figure 4G). *Aeromonas* and *Bacteroides* were lowest in T70, suggesting that moderate density does not favor the anaerobic or facultatively anaerobic niches that these genera occupy. *Bacillus* and *Pseudomonas* were highest in T70. Both genera are well-known for hydrolytic enzyme production and nicotine-degrading capacity [8], providing a microbiological explanation for the intermediate nicotine reduction observed in T70 (Figure 1D). *Sphingomonas* decreased with increasing density, while *Novosphingobium* showed the opposite trend. This reciprocal pattern suggests niche differentiation: *Sphingomonas* prefers more aerobic conditions (T60), whereas *Novosphingobium* is better adapted to lower-oxygen environments (T80). Both genera are involved in degrading aromatic compounds [46,47], so their density-dependent replacement may influence the profile of phenolic metabolites. For fungal genera, *Alternaria*, *Aspergillus*, *Mycosphaerella*, and *Botryosphaeria* reached their highest abundances in T70 (Figure 4H). The enrichment of these genera under moderate packing density suggests that microaerophilic and moderately humid conditions provide a favorable ecological niche for fungal taxa involved in extracellular enzyme secretion and secondary metabolism. In particular, *Aspergillus* is well known for its strong hydrolytic capacity, including the degradation of polysaccharides and proteins, which may contribute to the release of aroma precursors such as sugars, amino acids, and carotenoid-bound substrates [48]. *Alternaria* and *Botryosphaeria* have been reported to participate in the transformation of plant secondary metabolites, potentially influencing the formation of volatile aroma compounds [49].

LEfSe analysis identified distinct sets of microbial biomarkers for each treatment (Figure 4I). T60 biomarkers included *Arthrobacter*, *Pseudomonas*, *Weissella*, *Flavobacterium*, *Micrococcus*, and the yeast *Hanseniaspora*. These are predominantly aerobic or facultatively aerobic genera with rapid growth rates. *Pseudomonas* and *Arthrobacter* are well-documented nicotine degraders [8], explaining the lowest nicotine content in T60 (Figure 1D). The enrichment of these aerobic generalists reflects the high oxygen availability in loosely packed tobacco. T70 biomarkers included *Lactococcus*, *Leuconostoc*, *Bacillus*, *Enterococcus*, *Pediococcus*, along with the yeasts *Pichia* and *Aureobasidium*.

This assemblage is functionally significant for three reasons. First, the strong co-enrichment of multiple lactic acid bacteria (LAB) genera provides a direct microbiological explanation for the highest total ester content observed in T70 (Figure 2). LAB are prolific producers of esterases and alcohol acetyltransferases, enzymes that catalyze ester formation [50].

Second, the coexistence of LAB with *Bacillus* (a potent hydrolase producer) and *Enterobacteriaceae* (which generate diverse fermentation volatiles such as 2,3-butanediol and acetoin) suggests metabolic complementarity. *Bacillus* may liberate fermentable substrates from macromolecules, which LAB and Enterobacteriaceae then convert into aroma compounds [43,44,51].

Third, the enrichment of *Pichia* and *Aureobasidium* further enhances the aroma-producing capacity of the microbial community in the T70 treatment. These two fungal genera are well-documented to synthesize esters, higher alcohols, and a variety of volatile aromatic compounds that contribute to favorable sensory profiles [40,41]. This functional synergy, maximized at moderate density, explains why T70 outperformed both T60 and T80 in ester and ketone accumulation. T80 biomarkers included *Clostridium*, *Bifidobacterium*, *Propionibacterium*, *Megasphaera*, *Veillonella*, and so on. These are predominantly strict anaerobes. *Clostridium* species produce butyric acid and other short-chain fatty acids through fermentation [52], while *Propionibacterium* produces propionic acid [53]. The accumulation of these acidic metabolites is closely associated with the generation of rancid, cheesy, and pungent off-flavors in fermented products [54]. The enrichment of these genera in T80 provides a potential explanation for any harsh or unpleasant characteristics associated with high-density fermentation, although direct sensory evaluation would be required to confirm this.

### 3.4. Functional Predictions Reveal Density-Dependent Metabolic Reprogramming

**Figure 5:**
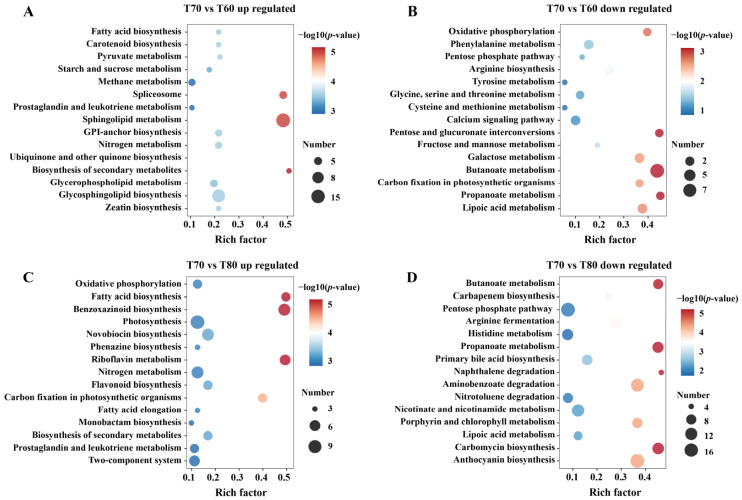
KEGG pathway enrichment analysis based on PICRUSt2 functional predictions. (**A**,**B**) Pathways upregulated and downregulated in T70 compared to T60, respectively. (**C**,**D**) Pathways upregulated and downregulated in T70 compared to T80, respectively. Bubble size represents the mean relative abundance of each pathway, and bubble color represents the significance level.

To link microbial community shifts to potential metabolic functions, KEGG pathway enrichment was compared among treatments. Compared to T60, T70 exhibited upregulation of pathways related to fatty acid biosynthesis, carotenoid biosynthesis, pyruvate metabolism, and starch and sucrose metabolism (Figure 5A). This pattern indicates that moderate density shifts metabolic investment from energy production toward anabolic processes that generate aroma precursors. The upregulation of fatty acid biosynthesis is particularly significant, as fatty acyl-CoAs are direct substrates for ester synthesis catalyzed by alcohol acyltransferases [55]. Coupled with the enrichment of LAB in T70 (Figure 4F), which are known to possess high alcohol acyltransferase activity [56], this functional prediction provides a mechanistic explanation for the highest total ester content observed in T70 (Figure 2).

The upregulation of carotenoid biosynthesis is noteworthy given that carotenoid cleavage products are key tobacco aroma compounds [34]. Pathways downregulated in T70 relative to T60 included oxidative phosphorylation, phenylalanine metabolism, the pentose phosphate pathway, and various amino acid metabolism pathways (Figure 5B). The downregulation of oxidative phosphorylation indicates reduced aerobic respiration in T70 compared to T60, consistent with the lower oxygen availability expected at moderate density. The broad suppression of amino acid metabolism pathways in T70 suggests that T60 supports more active catabolism of amino acids, which may contribute to the higher levels of phenylethyl alcohol and phenylacetaldehyde observed in T60 (Figure 2), as these aromatic metabolites are synthesized from phenylalanine via the Ehrlich pathway [38]. The downregulation of butanoate and propanoate metabolism in T70 relative to T60 indicates that T60 also supports some anaerobic fermentation activity, though less than T80.

The comparison between T70 and T80 also revealed pronounced functional divergence (Figure 5C,D). Pathways upregulated in T70 relative to T80 included oxidative phosphorylation, fatty acid biosynthesis, and various carbohydrate metabolism pathways (Figure 5C). This profile reflects a fundamentally aerobic metabolic regime in T70 that is largely suppressed in T80 due to severe oxygen limitation.

Pathways downregulated in T70 relative to T80 were dominated by anaerobic fermentation pathways (Figure 5D). The significantly enriched metabolic pathways in T80 encompass butanoate metabolism, the pentose phosphate pathway, arginine fermentation, histidine metabolism, propanoate metabolism, and methanogenesis. Of these pathways, the pronounced enrichment of butanoate metabolism in the T80 group is particularly notable. Butyrate is primarily synthesized by Clostridium and its affiliated genera [57], which were identified as T80 biomarkers (Figure 4F). The accumulation of butyrate and other short-chain fatty acids (SCFAs) can induce unpleasant rancid, cheesy, and sweaty off-odors in fermented products [58]. Similarly, the upregulation of histidine metabolism that drives histamine biosynthesis in T80 raises risks of biogenic amine accumulation. Such metabolites are responsible for undesirable off-flavors and may trigger adverse physiological effects in organisms [59]. The enrichment of propanoate metabolism aligns with the presence of *Propionibacterium* biomarkers in T80, as this genus produces propionic acid as a major fermentation end product [53]. Collectively, these functional predictions suggest that high-density fermentation (T80) favors the production of short-chain fatty acids and biogenic amines, which are generally undesirable in high-quality tobacco.

### 3.5. Co-Occurrence Network and Keystone Taxa Analysis Reveal Density-Dependent Microbial Interactions

**Figure 6:**
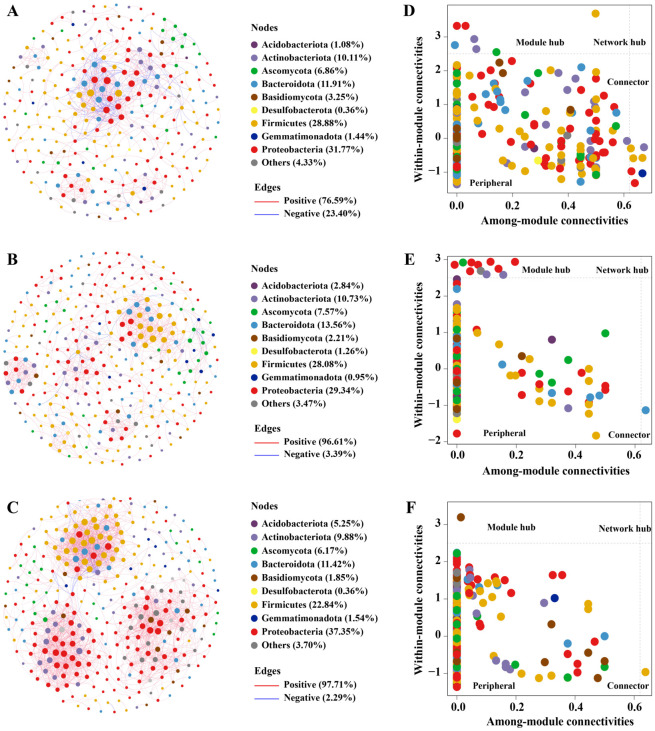
Co-occurrence network analysis of microbial communities under different packing densities. (**A**–**C**) Co-occurrence networks for T60, T70, and T80, respectively. Nodes are colored by phylum, and edge thickness represents correlation strength. (**D**–**F**) Zi-Pi analysis showing the topological roles of taxa in T60, T70, and T80. Nodes are classified as peripherals, connectors, module hubs, and network hubs.

To explore microbial coexistence patterns and community assembly mechanisms, we constructed co-occurrence networks for each packing density treatment (Figure 6A–C, Table 1). Network topology shifted markedly with packing density (Table 1). Total nodes increased modestly from T60 (277) to T80 (324), while edges increased dramatically from 705 (T60) to 827 (T70) to 2049 (T80), indicating that high-density packing forces tighter microbial coupling, likely as a compensatory response to oxygen limitation [60]. The proportion of positive edges increased from 76.6% in T60 to 96.6% in T70 and 97.7% in T80, while negative edges correspondingly decreased. This shift reflects a transition from competition-dominated interactions under aerobic conditions (T60) to cooperation-dominated interactions under oxygen-limited conditions (T70, T80), consistent with the stress-gradient hypothesis [61]. Firmicutes nodes decreased in T80, reflecting reduced fitness of many LAB and *Bacillus* members under severe oxygen limitation. Average degree and graph density were comparable between T60 and T70 but more than doubled in T80 (12.65 and 0.039, respectively), confirming ultra-dense connectivity at high density.

Notably, modularity peaked in T70 (0.82) compared to T60 (0.69) and T80 (0.68). High modularity indicates organization into distinct functional modules, which enhances stability and functional specialization [62]. The peak modularity in T70 aligns with its highest taxonomic diversity (Figure 4B,C) and supports the proposed metabolic complementarity among LAB, Bacillus, and Enterobacteriaceae. In contrast, the low modularity and near-absence of negative edges in T80 suggest a stress-adapted community with reduced functional redundancy. Thus, the modular, diverse, and balanced architecture of T70′s network underpins its superior chemical and aroma profiles.

Zi-Pi analysis was then performed to identify keystone taxa and understand how packing density reshapes network architecture (Figure 6D–F). Across all treatments, most nodes were peripherals, while keystone distribution varied markedly with density.

T60 harbored seven module hubs and eight connectors. Module hubs included members of Proteobacteria and Actinobacteriota, while connectors comprised Firmicutes, Actinobacteriota, and Proteobacteria. This decentralized structural pattern, characterized by a nearly equivalent abundance of hubs and connectors, demonstrates that the aerobic T60 microbial community forms a highly interconnected network. The frequent cross-module interactions within this network reflect competitive and cooperative microbial dynamics under oxygen-sufficient conditions [63]. T70 exhibited a strikingly different topology with 11 module hubs (predominantly Proteobacteria and Actinobacteriota) but only one connector. This shift from balanced hubs and connectors to hub dominated with minimal connectors indicates that moderate density promotes multiple locally influential taxa that operate with relative independence. The scarcity of connectors enables individual modules to act as semi-autonomous functional units, each structured around core hub taxa [64]. This compartmentalized architecture, consistent with the highest modularity observed for T70, has profound functional implications. Each module may correspond to an independent functional guild, and such modular organization supports parallel metabolic operations without mutual interference. The resulting metabolic complementarity further maximizes aroma synthesis efficiency [63]. T80 presented a radically simplified topology with only one module hub and one connector. The dramatic reduction in keystone taxa, from 15 in T60 to 12 in T70 to just 2 in T80, indicates that severe oxygen limitation collapses network complexity.

The loss of modular structure suggests the ultra-dense T80 network forces most taxa into a single, highly integrated cooperative web. Under extreme stress, niche differentiation breaks down, and all taxa are compelled to cooperate intensively for survival. While this architecture may be robust to node loss due to high connectivity, it lacks functional redundancy and may be vulnerable to cascading failure if the single hub or connector is lost [64].

### 3.6. Community Assembly Mechanisms Shift with Packing Density

**Figure 7:**
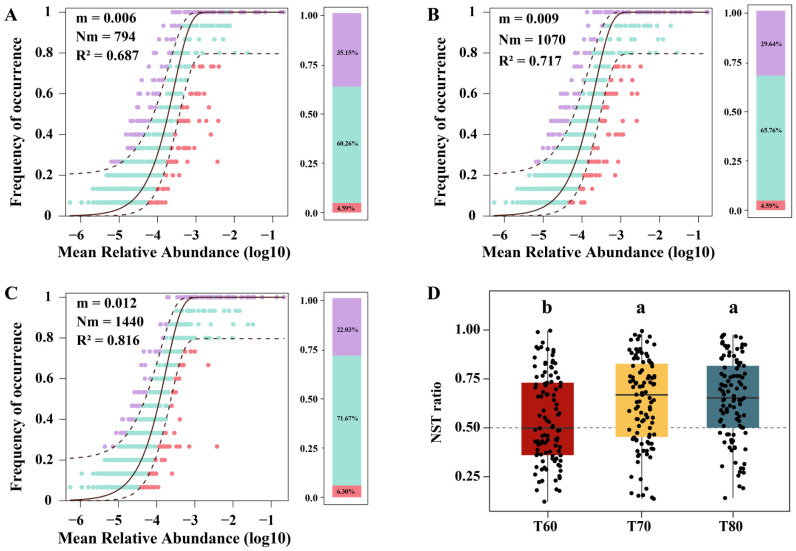
Neutral community model (NCM) fitting for microbial community assembly. (**A**–**C**) represent NCM for T60, T70, and T80, respectively. The solid line represents the best fit of the neutral model, and dashed lines represent 95% confidence intervals. (**D**) Normalized stochasticity ratio (NST) among treatments. Different letters indicate significant differences (*p* < 0.05).

The neutral community model (NCM) was fitted to elucidate the ecological processes governing microbial community assembly under different packing densities (Figure 7). The coefficient of determination (*R*^2^) of the NCM increased progressively with elevated packing density, indicating that stochastic processes play an increasingly dominant role as oxygen availability declines [18]. In T60, the relatively low *R*^2^ suggests that deterministic selection driven by environmental filtering under aerobic conditions plays a substantial role. Under oxygen-replete conditions, competitive exclusion favors the proliferation of fast-growing aerobic generalist taxa, resulting in microbial community structures that deviate from neutral model predictions [65]. In contrast, T80 exhibited the highest *R^2^*, indicating that stochastic processes dominate assembly. Severe oxygen limitation restricts survival to a limited set of obligate anaerobes, and their relative abundances become increasingly determined by random dispersal and ecological drift rather than niche-based selection [66]. T70 exhibited an intermediate *R*^2^, reflecting a mixed assembly regime where both deterministic and stochastic processes contribute. This balanced assembly mechanism is ecologically meaningful, as mixed assembly regimes are typically linked to higher biodiversity and functional stability in microbial ecosystems [67].

Consistent with this trend, both the migration rate (*m*) and metacommunity size (*Nm*) increased with rising packing density. This phenomenon indicates that physical confinement and enhanced contact frequency among tobacco particles facilitate microbial dispersal even under oxygen-deficient conditions. Elevated dispersal rates can improve local microbial diversity by introducing exogenous taxa that would otherwise be eliminated via competitive exclusion, which partially accounts for the high biodiversity detected in the T70 treatment [68].

We further calculated the normalized stochasticity ratio (NST) to assess the relative balance of stochastic versus deterministic processes. Notably, NST was significantly higher in T70 and T80 than in T60 (*p* < 0.05), confirming that stochasticity becomes increasingly influential as packing density increases. For T70, this high stochasticity operates within a diverse and modular network (Table 1), allowing multiple functional guilds to coexist without competitive exclusion. Such stochasticity-mediated coexistence, coupled with moderate deterministic environmental filtering, establishes favorable conditions for maximizing both taxonomic diversity and functional complementarity of communities [68]. In contrast, T60′s lower NST reflects stronger deterministic filtering, which reduces diversity by favoring a subset of aerobic generalists. T80, despite having high NST, operates within a severely constrained species pool, limiting functional diversity. Collectively, the T70 group achieves a unique optimal balance between stochastic and deterministic processes. The joint regulation of these two processes shapes functionally robust microbial communities, which ultimately accounts for the superior aroma characteristics observed in the T70 system.

More broadly, these findings are consistent with the growing scientific interest in microbial ecology as a key framework for understanding and managing biological production systems [69]. Microbial communities are increasingly recognized as important drivers of food and agricultural processes because they regulate substrate transformation, nutrient cycling, plant performance, product quality, and system stability [70]. In both natural and managed ecosystems, shifts in microbial community composition and diversity can influence ecosystem functions and production outcomes [71]. Similarly, in fermentation systems, microbial community structure, functional traits, and interspecies interactions directly influence metabolite formation, flavor development, safety, and product consistency [72]. Therefore, identifying how a controllable processing factor such as packing density regulates microbial assembly and interaction networks not only improves our understanding of tobacco fermentation, but also contributes to the broader development of microbiome-guided strategies for quality control and sustainable bioprocessing.

## 4. Conclusions

This study reveals that packing density fundamentally shapes tobacco fermentation quality by modulating microbial community assembly, metabolic function, and ecological interactions. Moderate density (70%) achieves the most balanced chemical and aroma profiles by enriching aroma-producing taxa and promoting a modular network architecture that enables metabolic complementarity. These findings establish a mechanistic framework linking a physical processing parameter to product quality through microbial ecology. Future studies should validate these findings at an industrial scale and explore metatranscriptomic approaches to confirm the expression of key metabolic pathways.

## Figures and Tables

**Table 1:** Topological indices of co-occurrence networks.

Network	Element	T60	T70	T80
Node	Total	277	317	324
	Acidobacteriota	1.08%	1.26%	0%
	Actinobacteriota	10.11%	10.73%	9.88%
	Ascomycota	6.86%	7.57%	6.17%
	Bacteroidota	11.91%	13.56%	11.42%
	Basidiomycota	3.25%	2.84%	5.25%
	Desulfobacterota	0.36%	0.95%	1.54%
	Firmicutes	28.88%	28.08%	22.84%
	Gemmatimonadota	1.44%	2.21%	1.85%
	Proteobacteria	31.77%	29.34%	37.35%
	Others	4.33%	3.47%	3.70%
Edge	Total	705	827	2049
	Positive	76.59%	96.61%	97.71%
	Negative	23.40%	3.39%	2.29%
Topological parameters	Average degree	5.09	5.22	12.65
	Average path length	3.62	3.64	3.28
	Graph diameter	12	12	11
	Graph density	0.018	0.017	0.039
	Clustering coefficient	0.51	0.56	0.57
	Modularity	0.69	0.82	0.68

## Data Availability

The original contributions presented in this study are included in the article. Further inquiries can be directed to the corresponding author.
